# Maternal BMI and Diet Quality Modulate Pregnancy Oxidative and Inflammatory Homeostasis

**DOI:** 10.3390/nu17162590

**Published:** 2025-08-09

**Authors:** Chiara Mandò, Chiara Novielli, Anna Maria Nuzzo, Francesca Parisi, Laura Moretti, Fabrizia Lisso, Alberto Revelli, Valeria M. Savasi, Arianna Laoreti, Gaia M. Anelli, Alessandro Rolfo, Irene Cetin

**Affiliations:** 1 Department of Biomedical and Clinical Sciences, Università degli Studi di Milano, 20157 Milan, Italy; chiara.mando@unimi.it (C.M.); francesca.parisi@unimi.it (F.P.); fabrizia.lisso@asand.it (F.L.); valeria.savasi@unimi.it (V.M.S.); gaia.anelli@unimi.it (G.M.A.); 2 Department of Woman, Mother and Neonate, “Vittore Buzzi” Children’s Hospital, ASST Fatebenefratelli Sacco, 20154 Milan, Italy; arianna.laoreti@asst-fbf-sacco.it (A.L.); irene.cetin@unimi.it (I.C.); 3 Department of Surgical Sciences, Gynecology and Obstetrics 2U, S. Anna Hospital, University of Turin, 10126 Turin, Italy; a.nuzzo@unito.it (A.M.N.); l.moretti@unito.it (L.M.); alberto.revelli@unito.it (A.R.); alessandro.rolfo@unito.it (A.R.); 4 S.C. Ostetricia, Foundation IRCCS Ca’ Granda Ospedale Maggiore Policlinico, 20122 Milan, Italy; 5 Technical Scientific Association of Food, Nutrition and Dietetics (ASAND), 95128 Catania, Italy; 6 Department of Clinical Sciences and Community Health, Dipartimento di Eccellenza 2023–2027, Università degli Studi di Milano, 20122 Milan, Italy

**Keywords:** pre-pregnancy body mass index, maternal obesity, maternal overweight, maternal nutrition, dietary patterns, oxidative stress, inflammation, pregnancy, perinatal outcomes, individualized nutritional strategy

## Abstract

**Background/Objectives:** Maternal nutrition and pregestational BMI are critical determinants of pregnancy outcomes. This prospective multicenter observational study investigated the interplay between prepregnancy BMI, dietary patterns, and oxidative/inflammatory status in 153 Italian healthy pregnant women with normal weight (NW), overweight (OW), or obesity (OB). **Methods:** Detailed clinical, biochemical, placental, and neonatal data were measured at third trimester and delivery. Dietary intake was assessed via a validated questionnaire, and dietary patterns were derived using principal component analysis. **Results:** OW and OB women had significantly higher levels of inflammatory (CRP, hepcidin) and oxidative stress biomarkers (DNA/RNA damage, catalase activity) than NW. Multivariate models confirmed independent associations between BMI and these biomarkers (CRP: β = 0.297, *p* = 0.000; hepcidin: β = 1.419, *p* = 0.006; DNA/RNA damage: β = 409.9, *p* = 0.000; catalase activity: β = 1.536, *p* = 0.000). Superoxide dismutase activity and total antioxidant capacity were not associated with BMI. Nutritional intake across BMI groups was largely suboptimal relative to national recommendations, with insufficient levels of polyunsaturated fats and key micronutrients. Four dietary patterns were identified, with adherence varying by BMI. A “prudent-style” pattern (high plant, low animal) was positively associated with gestational age (β = 0.243, *p* = 0.033) and inversely with neonatal head circumference (β = −0.414, *p* = 0.050). A “Western-like” pattern (high sugars, snacks, animal fats) was linked to reduced maternal ferritin (β = −2.093, *p* = 0.036) and increased neonatal head circumference (β = 0.403, *p* = 0.036). However, not all deviations from the “prudent-style” pattern were metabolically equivalent: while Pattern 3 (high-protein, carbohydrate) may offer partial protective effects, Pattern 4 (moderate protein/plant/sugar) displayed elements of nutritional imbalance with signs of placental inefficiency (β = −0.384, *p* = 0.023). **Conclusions:** These findings underscore the dual impact of maternal BMI and diet quality on oxidative-inflammatory balance and perinatal outcomes, supporting the need for early, individualized nutritional strategies in pregnancy. This is further emphasized by the variability in dietary adherence across BMI categories.

## 1. Introduction

Entering pregnancy with overweight or obesity poses significant health risks for mother and baby, both in the short and long term, extending to the child’s future health as an adult. Increasing evidence has confirmed the fetal programming theory, which suggests that the intrauterine period is a time of remarkable plasticity for the individual [1,2,3,4]. During this phase, the fetus responds to external stimuli—primarily maternal nutrition—shaping its genetic potential according to the surrounding environment.

In this framework, an excess of maternal adipose tissue plays a key role, being linked to an increased pro-inflammatory response and low-grade chronic inflammation, which, in a self-perpetuating negative feedback loop, contributes to cellular oxidative stress, impaired metabolic function, and cellular senescence [5,6,7]. During pregnancy, this can lead to a state of increased inflammaging at both the systemic and intrauterine levels. When combined with oxidative stress resulting from mitochondrial dysfunction and/or an imbalance between reactive oxygen species and antioxidant defenses, this condition can potentially impair placental function and adversely affect fetal development [8,9,10,11].

In this context, an unbalanced maternal intake of macro- and micronutrients can further exacerbate the negative effects of excessive adipose tissue. During pregnancy, the proper intake of macro- and micronutrients is crucial for healthy fetal programming [12,13,14,15]. Moreover, growing evidence suggests that dietary patterns, rather than individual nutrients, are key factors in successful pregnancies, promoting the long-term health of both the baby and the mother. A “prudent-style” diet, such as the Mediterranean diet, characterized by high fiber content, a lipid profile rich in monounsaturated and polyunsaturated fatty acids, and by an abundance of other components such as polyphenols, has proven to be particularly protective, significantly reducing overall and cardiovascular mortality [16,17,18,19]. Indeed, dietary patterns such as the Mediterranean diet are rich in nutrients that work synergistically, acting on cellular mechanisms that stimulate mitochondrial biogenesis and reduce inflammation and oxidative stress, modulating parameters such as blood pressure, lipid profile, body weight, and fasting blood glucose [19,20]. Conversely, these dietary patterns avoid animal nutrient sources and are low in processed food rich in salt, saturated and trans fatty acids, sugar, and other nutrients that have demonstrated detrimental effects on health. High-fat and ultra-processed foods, typically found in the Western diet, are associated with characteristics closely linked to cardiovascular risk and mortality [21,22,23,24,25,26,27]. However, since the 1960s, adherence to the Mediterranean diet has progressively decreased worldwide, in favor of a Western-like diet, representing a significant risk for the health of both mothers and children in the short and the long term [23,24,25].

Therefore, it is essential to clarify the dietary patterns followed by pregnant women and determine any related oxidative and inflammatory alterations, also in relation to their pre-pregnancy body mass index (BMI), in order to identify mechanisms potentially harmful to fetal programming and to the future health of the mother and her child, laying the groundwork for the implementation of national and international guidelines for proper nutrition during pregnancy.

In this study, nutritional, inflammatory, and oxidative parameters were evaluated in Italian pregnant women with normal weight, overweight, or obesity, identifying dietary pattern adherence during pregnancy and associations with obstetric, placental, and neonatal parameters. We tested the hypothesis that maternal dietary patterns and pregestational BMI jointly influence maternal oxidative and inflammatory status, with downstream effects on placental and neonatal outcomes.

## 2. Materials and Methods

### 2.1. Study Design and Population

This study is part of the project “Epigenetic impact of maternal obesity and nutritional status. Nutritional/lifestyle intervention for the improvement of pregnancy outcomes (EPI-MOM)” funded by the Italian Ministry of Health (Ricerca Finalizzata, RF-2016-02362165).

This multicenter project has been conducted in the obstetric units of two centers in Italy, the “Vittore Buzzi” Children’s Hospital (ASST Fatebenefratelli Sacco) in Milan and the AOU Città della Salute e della Scienza—Sant’Anna University Hospital in Turin.

The protocol was approved by the Hospital ethical committee “Comitato Etico Milano Area 1” (Prot. N. 17739/2018) and by O.I.R.M.-Sant’Anna Hospital and “Ordine Mauriziano di Torino” ethical committee (Prot. N. 0066426/2019). The study was conducted in accordance with the Declaration of Helsinki and in compliance with all current Good Clinical Practice guidelines, local laws, regulations, and organizations. All participants provided their informed consent to collect personal data and biological samples.

This multicenter prospective cohort study was conducted among pregnant women stratified according to pregestational BMI.

Women were enrolled between 30 + 0 and 36 + 6 gestational weeks during III trimester routine checkup visits at the antenatal clinic. Clinical data and biological samples were collected by the clinical and research staff participating in the study, at both enrollment (T0) and delivery (T1).

The study population consisted of healthy Caucasian women aged 18–40 years, with a pregestational BMI between 18.5 and 40 kg/m^2^ and a singleton spontaneous pregnancy. Exclusion criteria were any maternal chronic comorbidity (i.e., chronic hypertension, autoimmune diseases, pregestational diabetes), pregnancy complications in present or previous pregnancies (e.g., GDM, preeclampsia, infections, or congenital/genetic abnormalities), and any pharmacological therapy in pregnancy, including metformin and insulin.

Participants were divided into three groups based on their pregestational BMI:

- Normal weight (NW): 18.5 kg/m^2^ ≤ pregestational BMI < 25 kg/m^2^;
- Overweight (OW): 25.0 kg/m^2^ ≤ pregestational BMI < 30 kg/m^2^;
- Obese (OB): 30.0 kg/m^2^ ≤ pregestational BMI ≤ 40 kg/m^2^.

### 2.2. Clinical Data

Maternal age, pregestational weight, and BMI were recorded, together with information on folic acid/iron/multivitamin supplementation. The supplements used were from different commercial brands, but all contained, at a minimum, 400 µg of folic acid (folic acid supplementation), 80–100 mg of elemental iron (iron supplementation), and the most common vitamins typically included in prenatal formulations (multivitamin supplementation). Regarding duration, folic acid was generally taken from the first trimester, while multivitamin and iron supplements were started either in the first or second trimester.

The following data were collected at both T0 and T1: gestational age; maternal weight gain, hemoglobin concentration, hematocrit. At T0, ferritin, vitamin D, and glucose blood concentration values were also recorded. Mode of delivery, neonatal sex, weight, ponderal index, head circumference, and umbilical arterial pH data were collected at delivery.

### 2.3. Blood Collection, Placental Biometric Measurements, Inflammatory and Oxidative Markers Assessment

At T0, maternal venous blood was withdrawn from a radial vein and collected in EDTA or serum-separator tubes. Samples were centrifuged at 4000× *g* for 10 min at 4 °C; plasma or serum were separated from cellular components and stored at −80 °C until use.

Placentas were weighed without membranes and cord, and after cleaning excess blood. Placental area was estimated by measuring diameters and calculating the area of an ellipse (D × d × π/4). Placental efficiency was calculated as the ratio between neonatal weight and placental weight [28].

Levels of the inflammatory biomarkers hepcidin and C reactive protein (CRP) were evaluated in maternal serum, while activities of the antioxidant enzymes catalase (CAT) and superoxide dismutase (SOD), as well as total antioxidant capacity (TAC) and DNA/RNA oxidative damage, were measured in maternal plasma, all sampled at third trimester.

Hepcidin. Serum levels of the bioactive peptide hepcidin-25 (ng/mL) were determined using a commercially available ELISA kit (DRG Diagnostic GmbH, Marburg, Germany) according to the manufacturer’s instruction. The kit was based on the principle of competitive binding where endogenous hepcidin of sample competed with the added hepcidin-biotin conjugate for binding to the coated antibody. Absorbance was determined at 450 nm using a microplate reader.

C reactive protein (mg/L) serum level was determined using a commercially available ELISA kit (Alpha Diagnostic International, San Antonio, TX, USA) according to the manufacturer’s instruction. The assay was based on simultaneous binding of human CRP from samples to two antibodies, one immobilized on the microtiter well plate, and the other conjugated to the enzyme horseradish peroxidase. Absorbance was measured on a microtiter well ELISA reader at 450 nm, and concentrations of CRP in samples were read off the standard curve.

Catalase and superoxide dismutase enzyme activities on plasma were determined using commercially available kits (Cayman Chemical, Ann Arbor, MI, USA) and following the manufacturer’s instructions. Briefly, CAT activity was determined by measuring catalase peroxidative function based on the reaction between CAT and methanol in the presence of an optimum concentration of hydrogen peroxide. Formaldehyde was measured spectrophotometrically at 540 nm using 4 amino-3-hydrazino-5-mercapto-1,2,4-triazole. Results are expressed in nmol/min/mL. Total SOD activity was measured by reduction of cytochrome C by superoxide (O_2_^•−^) radicals monitored spectrophotometrically at 450 nm using the xanthine-xanthine oxidase system. Results are expressed in U/mL.

Total antioxidant capacity was assessed on plasma using the Cayman Chemical Antioxidant Assay, which measures the ability of antioxidants to inhibit ABTS oxidation. Absorbance was recorded at 750 nm using an ELISA microplate reader.

Oxidative damage to DNA/RNA. Maternal plasma was assayed for oxidized guanine species (8-OHG, 8-OH-dG, hydroxyguanosine), released during the repair process following DNA/RNA damage. The DNA/RNA Oxidative Damage (High Sensitivity) ELISA Kit (Cayman Chemical) was used, following the manufacturer’s instructions. Plasma samples were assayed at 1:50 dilution in duplicate. Concentrations were calculated with the data analysis tool supplied by the manufacturer (https://www.myassays.com/8-hydroxy-2-deoxy-guanosine.assay (accessed on 10 January 2024)).

### 2.4. Dietary Intake Assessment

At T0, participants completed a validated semi-quantitative food frequency questionnaire (FFQ) covering dietary intake over the preceding 3 months. The FFQ, originally developed for the Italian population, included 192 items and was slightly adapted to reflect recent eating habits and preferences, as previously detailed [29]. Individual daily intakes of energy, macronutrients, and micronutrients were then calculated by dietitians and nutritionists for each participant. For each item, consumption frequency and portion size were entered in a spreadsheet together with the bromatological composition, obtained from the Food Composition Database for Epidemiological Studies in Italy (BDA, www.bda-ieo.it/ (accessed on 28 April 2021)) and the Food Composition Tables of the Council for Agricultural Research and Analysis of Agricultural Economics (CREA, www.crea.gov.it (accessed on 28 April 2021)), as previously reported [13]. An example of calculation of the daily amount of energy and macro- and micronutrients is reported in the Supplementary Materials.

Mean intake levels were also compared with the Italian recommendations outlined in the LARN (“Reference Intake Levels of Nutrients and Energy for the Italian Population”) [30]. In this document, pregnancy-specific reference values are reported for proteins, lipids, minerals, vitamins, and water. The energy reference level (2646 kcal) was derived from the LARN guidelines for Italian women aged 30–59 years, with an average height of 1.65 m (similar to our study population), an average weight of 61.3 kg, and an average daily physical activity level of 1.6, corresponding to moderate activity. This value was adjusted by adding the energy cost of pregnancy during the third trimester (496 kcal/day), based on a gestational weight gain (GWG) of 12 kg in previously normal-weight women.

Additionally, the 192 food items from the FFQ were grouped into 15 food categories—dairy, cereals, vegetables, legumes, potatoes, meat, fish, eggs, fruit, nuts, vegetable fats, animal fats, sauces, sweets, and non-alcoholic beverages, based on their origin and similarities in nutrient composition. This grouping strategy is consistent with approaches adopted in previous studies that used principal component analysis (PCA) to derive dietary patterns [29,31,32].

### 2.5. Statistical Analysis

Analyses were performed using SPSS Statistics v.29 (IBM; Armonk, NY, USA). Data were tested for outliers; *p*-value < 0.05 was considered statistically significant.

Data were compared among the three BMI categories using one-way ANOVA (and Tukey’s test for post-hoc analyses) or Kruskal–Wallis test (and pairwise comparisons with Bonferroni’s correction), according to data distribution assessed with the Kolmogorov–Smirnov test. Frequencies (parity, supplementation, anemia, adherence to weight gain recommendations, mode of delivery, neonatal sex, adherence to dietary patterns) were compared among the three BMI groups with Chi-square test or Fisher’s exact test.

Dietary patterns were extracted from reliable FFQs by using the PCA, which aggregates specific food groups into complex dietary patterns according to the degree of the inter-correlation, as detailed in Anelli et al. 2022 [29]. Only dietary patterns with eigenvalues ≥ 1.1 were extracted, thus reducing the bias of multiple testing. After performing the PCA, each food group automatically receives a factor loading representing the strength of correlation to the extracted dietary patterns. Finally, when performing the PCA, all women are automatically assigned a component score for each extracted dietary pattern, representing their adherence to that specific dietary pattern. Dietary pattern adherence (component scores) was compared between the study groups by using the Kruskal–Wallis test. To estimate associations between BMI groups, maternal dietary pattern adherence (component scores), biomarker concentrations, and delivery outcomes, general linear and binary logistic regression models adjusted for confounding factors (maternal age, pregestational BMI, parity, supplementation; gestational age at T0, gestational weight gain at T0; energy; gestational age at T1, gestational weight gain at T1, neonatal sex) were estimated.

The analyses and data presented in this manuscript refer to secondary aims of the broader research project. As such, a specific sample size calculation was not performed for these outcomes, which were explored within the framework of a real-world observational study.

## 3. Results

A total of 85 NW, 48 OW, and 29 OB women were enrolled. Six NW and three OW women discontinued the study due to personal reasons. Reasons for discontinuation included participant withdrawal of consent, lack of availability or follow-up, and logistical difficulties during the COVID-19 emergency. Therefore the described population included 79 NW, 45 OW, and 29 OB pregnant women.

### 3.1. Maternal, Placental, and Neonatal Data

Table 1 reports maternal data (general, at third trimester, and at delivery) and placental and neonatal data.

Notably, the frequency of not supplemented women tended to be higher in both OW and OB than in controls, and in OB, a larger percentage of women needed iron supplementation.

At T0, OB women had gained on average significantly less weight than those in both the NW and OW groups, although there was considerable variability among individuals. Hemoglobin and hematocrit levels were higher in OB than in OW women, but no significant differences in anemia frequency were found among groups. Moreover, glucose levels were significantly higher in both OW and OB women compared to NW women, but they remained below the diagnostic cut-off for gestational diabetes, according to the study inclusion criteria.

At delivery, gestational weight gain (GWG) remained on average significantly lower in the OB group compared to both the NW and OW groups. GWG was also assessed based on adherence to the Institute of Medicine (IOM) recommendations, which are stratified by pregestational BMI. The proportions of women whose GWG fell below, within, or above the recommended ranges were calculated for each BMI category, revealing significant overall differences. Specifically, a substantial proportion of OW and OB women gained more weight than recommended. OW women had lower hemoglobin and hematocrit levels and showed a higher prevalence of anemia compared to both NW and OB groups.

Finally, it is noteworthy that placental characteristics were not significantly different among the three BMI groups, but OW and OB mothers showed higher placental weight and lower neonatal/placental weight ratios than controls, and placental area was less extended in the OB group, suggesting lower placental efficiency with increasing BMI.

### 3.2. Inflammatory and Oxidative Markers at Third Trimester

Results of comparison among the three BMI groups are reported in Table 2. Hepcidin and CRP levels, as well as catalase activity and DNA/RNA oxidative damage, increased with BMI and were significantly higher in both OW and OB women compared to controls. No differences among the three BMI groups were found for SOD activity and total antioxidant capacity.

### 3.3. Association of Maternal Markers and Clinical Maternal, Placental, and Neonatal Data

Table 3 shows the significant results from generalized linear models estimating the associations between maternal inflammatory/oxidative biomarkers at T0 and clinical data.

Interestingly, even after adjusting for confounding factors, pregestational BMI was significantly associated with hepcidin and CRP levels, catalase activity, and DNA/RNA oxidative damage, thereby strengthening the evidence observed in the group comparisons (Table 2).

Hepcidin levels were positively associated with neonatal ponderal index. CRP concentration was negatively associated with neonatal head circumference. Positive associations were found between catalase activity and neonatal weight, and between SOD activity and neonatal ponderal index.

Notably, supplementation with multivitamins was positively associated with total antioxidant capacity.

Regarding placental biometric parameters, catalase activity was significantly associate with placental weight.

### 3.4. Energy and Nutrient Daily Intake

Table 4 reports the mean daily intake levels for energy, macro-, and micronutrients among the three population groups (NW, OW, OB), calculated from the Food Frequency Questionnaires administered at third trimester of pregnancy and related to the previous 3 months.

Energy intake was not significantly different among groups.

Among macronutrients, no significant differences were found in protein and carbohydrate intake levels, except for fructose intake, which was significantly different among the three groups (*p* = 0.048), although statistical significance was not retained in the pairwise comparisons (OW-NW; OB-NW; OB-OW).

Conversely, lipid intake showed differences among groups. Indeed, OW women showed a significantly higher intake of total lipids compared to OB and of vegetable lipids compared to both OB and NW. Consistently, the intakes of monounsaturated fatty acids (MUFA) and oleic acid were significantly lower in OB compared to OW, while arachidic acid was higher in OW than in NW.

Among micronutrients, vitamins E and K showed significantly different intakes among groups: intake of vitamin E was lower in OB than in OW women, whereas vitamin K intake was lower in both OW and OB groups compared to NW.

**Figure 1:**
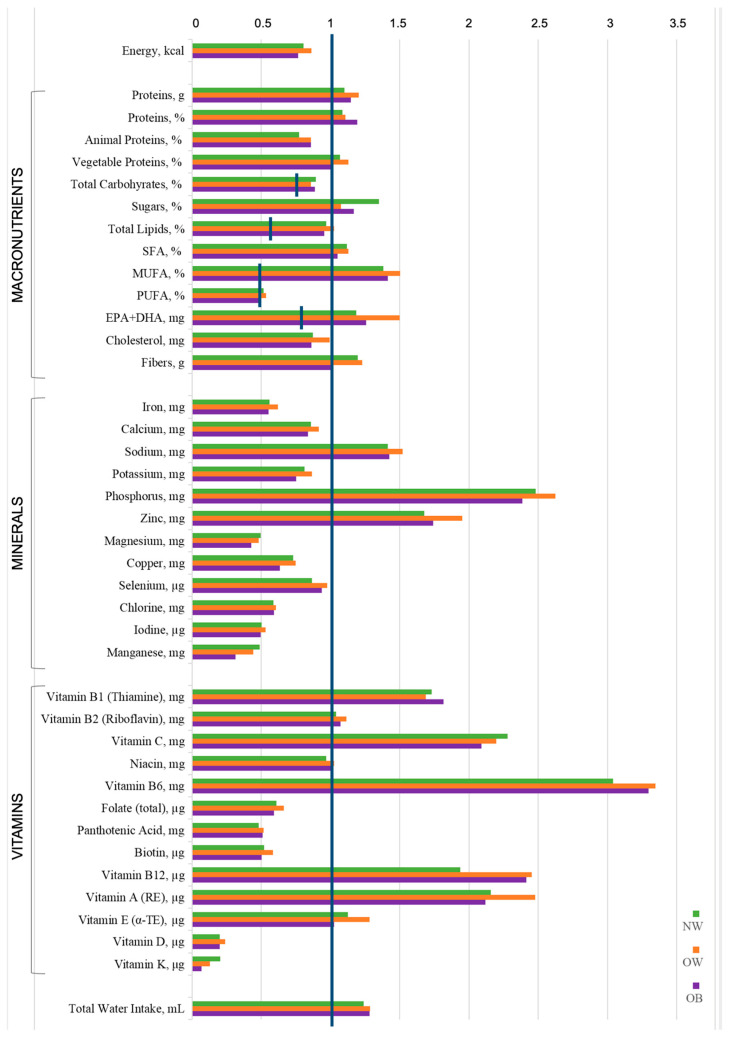
Histogram representation of the average intake values of energy and nutrients compared to LARN (Italian Recommended Nutrient Intake Levels [30]). The blue line represents 100% of the recommendations. When LARN values are expressed as ranges, the upper continuous line refers to the upper limit of the range, while the lower discontinuous lines refer to the lower limit of the range. SFA = saturated fatty acids; MUFA = monounsaturated fatty acids; PUFA = polyunsaturated fatty acids; EPA = eicosapentaenoic acid; DHA = docosahexaenoic acid; RE = retinol equivalents; α-TE = α-tocopherol equivalents; Total Water Intake = drinking water, and water assumed from food and beverages.

The mean intake levels for each BMI category (NW, OW, OB) were then compared to the Reference intake levels of nutrients and energy for the Italian population (LARN [30]) (Figure 1).

The energy requirement was not reached in any of our study categories.

Conversely, the mean intake of total proteins, both as percentage of total energy and in grams, exceeded recommendations in each group of pregnant women. The percentage of total carbohydrates was adequate (falling within the reference range of 45–60% of total energy intake), but the intake of sugars was excessive. The average percentage of total lipids was similar to the recommended levels, but intakes of both saturated (SFA) and monounsaturated (MUFA) fatty acids exceeded the recommended maximum percentages, while polyunsaturated fatty acids (PUFA) were assumed in markedly lower amounts than the reference levels across the entire study population. Concerning specific lipids, EPA and DHA intakes surpassed LARN recommendations, and cholesterol levels were below the established maximum.

Several deficiencies in mineral intakes were observed, regardless of BMI category. Indeed, only sodium, phosphorus, and zinc exceeded the reference values. Vitamin intake was highly irregular. Vitamin K and vitamin D were substantially insufficient to meet recommendations in all population groups; similarly, folates, pantothenic acid, and biotin did not reach adequate levels. Conversely, vitamins B1, B6, B12, C, and A surpassed reference values across all BMI categories.

Finally, water consumption was adequate in all women.

### 3.5. Dietary Patterns

After grouping FFQ items into 15 food categories, four dietary patterns were extracted using principal component analysis. These four patterns overall explained 49% of maternal dietary intake variance in the entire study population (Table 5).

Specifically:

- Pattern 1 (high plant, low animal: “Prudent-style”) was characterized by high adherence to legumes, nuts, vegetables, and fruit, and low adherence to sauces.
- Pattern 2 (ultra-processed food, high animal: “Western-like”) showed high adherence to sugar and snacks, animal fats, cereals, and dairy products.
- Pattern 3 (high protein and carbohydrates: “Moderately beneficial”) featured high fish, meat, and potatoes.
- Pattern 4 (moderate protein, moderate plant, moderate sugar: “Moderate-mixed”) was defined by high adherence to eggs, fruits, vegetable fats, and non-alcoholic beverages (such as sugar-sweetened beverages, artificially sweetened drinks, fruit juices, coffee, tea).

In the comparison among NW, OW, and OB women, adherence to Pattern 1 differed significantly among groups (Chi-square test: *p* = 0.029), with adherence decreasing as BMI increased.

OW women showed a significantly higher mean adherence value to Pattern 4 compared to NW women.

Multi-adjusted generalized linear models were performed to evaluate the associations between adherence to dietary patterns (component scores) and maternal blood markers at 3rd trimester and neonatal and placental data (Table 6).

Pattern 1 showed a positive association with gestational age at delivery and a negative association with neonatal head circumference; interestingly, the latter was positively associated with Pattern 2. A negative association was observed between Pattern 2 and ferritin levels. Pattern 3 and Pattern 4 were respectively associated with total antioxidant capacity (positively) and hemoglobin concentration (negatively).

Pattern 4 was also positively associated with placental weight and negatively with the neonatal/placental weight ratio.

Furthermore, among women adhering to Patterns 3 and 4, maternal energy intake was negatively associated with placental weight or area and positively associated with the neonatal/placental weight ratio.

## 4. Discussion

This study explored the relationship between dietary patterns during pregnancy and maternal oxidative-inflammatory status, as well as obstetric, placental, and neonatal outcomes in women with different pregestational BMI. The results confirmed and extended current evidence by integrating nutritional, biochemical, and obstetric data, suggesting how both excess maternal adiposity and suboptimal diet quality may contribute to a pro-inflammatory and pro-oxidant intrauterine environment, potentially affecting maternal health and fetal development.

### 4.1. Study Population and Maternal Blood Biomarkers

Despite the overall good health status of the study population and the absence of conditions known to influence the outcomes under investigation, numerous alterations were nonetheless identified, clearly associated with pregestational BMI and individual dietary intake and patterns.

The present findings showed that both overweight and obese women exhibited significantly higher circulating levels of inflammatory markers (hepcidin and CRP) and increased oxidative stress (DNA/RNA oxidative damage and catalase activity) compared to normal-weight women, corroborating recent evidence linking excess maternal adiposity to chronic low-grade inflammation and redox imbalance during gestation [11,33,34,35,36]. Importantly, the associations between pregestational BMI and these biomarkers remained significant even after adjusting for confounding variables, supporting the hypothesis of a potential independent contribution of excess adiposity, although no causal inferences can be drawn from this observational design. These results align with the growing body of evidence supporting the concept of maternal “inflammaging” induced by excess adiposity [8,37,38,39,40] that disrupts redox balance, immunomodulation, and endothelial function—which are increasingly recognized as key modulators of placental function and fetal programming—with consequences for both the mother and fetus [41,42,43,44,45,46,47,48,49,50]. Emerging evidence provides mechanistic support for the association between nutrient deficiencies, oxidative stress, and abnormal placentation and fetal growth. For instance, studies by Jansson & Powell [51,52,53] showed that maternal undernutrition or obesity alters placental mTOR signaling and amino acid transport, predisposing to fetal growth restriction or overgrowth via dysregulated nutrient sensing and NF κB pathway activation. Furthermore, human and animal studies [54,55,56] demonstrate that oxidative stress impairs trophoblast invasion and spiral artery remodeling, mediated via NF κB upregulation and diminished Nrf2-driven antioxidant defense, ultimately reducing placental perfusion, functionality, and nutrient delivery. Consistent with this, recent reviews [57,58] highlight how dietary patterns influence placental reactive oxygen species generation and anti-inflammatory pathways (e.g., Nrf2), suggesting that antioxidant micronutrients (e.g., vitamins, polyphenols, omega-3) modulate these signaling cascades toward improved placental structure and function.

The frequency of not supplemented women was higher in both the OW and OB groups than in controls, and a larger percentage of OB women needed iron supplementation, which might explain their higher hemoglobin concentration. Notably, in the total population, multivitamin supplementation was positively associated with antioxidant capacity, reinforcing the protective role of micronutrients in counteracting the pro-oxidant environment typical of obesity [14,59,60,61,62,63]. Nevertheless, OW and OB women exhibited higher levels of oxidative stress markers, highlighting the substantial increase in oxidative stress associated with excess adipose tissue.

The observed associations between hepcidin and neonatal anthropometrics further suggest that inflammation and, possibly, iron homeostasis may be involved in the modulation of fetal growth and body composition. Elevated CRP was negatively associated with neonatal head circumference, while catalase activity was positively related to both neonatal and placental weight, and SOD activity with neonatal ponderal index. These findings reinforce the role of redox and inflammatory balance in mediating maternal–fetal nutrient exchange and tissue development [64,65,66,67,68,69].

In terms of gestational weight dynamics, OW and OB women presented higher weight at delivery, but women with pregestational obesity gained significantly less weight during pregnancy. Despite this, a considerable proportion of OW and also OB women exceeded the Institute of Medicine (IOM) recommendations. As shown, maternal levels of inflammatory and oxidative markers were increased in women with higher BMI, suggesting that even moderate weight gain when pregestational BMI is elevated cannot counteract the negative effect of excessive pre-pregnancy adiposity, playing a critical role in determining maternal and neonatal risk profiles [9]. This supports calls to revisit current GWG thresholds in these populations [70,71,72,73]. If lower GWG thresholds were adopted for OW and OB women, an even greater proportion of the cohort in our study would fall into a higher-risk category—potentially explained, at least in part, by the inflammatory and oxidative imbalances that were observed.

### 4.2. Dietary Analysis and Association with Biomarkers and Clinical Data

The dietary analysis revealed that while total energy intake did not differ significantly among BMI groups, the composition and quality of macronutrients and micronutrients partially varied. Specifically, overweight women showed a higher total lipid intake, confirmed by increased consumption in specific categories such as monounsaturated fatty acids (MUFA), oleic acid, and vegetable fats. Moreover, women with pregestational obesity showed a significantly lower intake of vitamin E (α-tocopherol), which is known to contribute to antioxidant defense and anti-inflammatory activity and is associated with positive birth outcomes [74,75,76], while both OW and OB showed decreased vitamin K intake, whose deficiency during pregnancy can lead to severe maternal and neonatal hemorrhagic complications [77,78,79].

Importantly, regardless of intake differences between BMI groups, most women in the study did not comply with the Italian dietary recommendations for pregnant women [30], indicating an overall poor diet quality. Mean energy intake across all BMI categories fell below the recommended levels. Notably, the intake of polyunsaturated fatty acids, as well as several minerals and vitamins, was significantly lower than the recommended levels. Such inadequacies highlight the need for nutritional counseling based on not only quantity but also quality, particularly in populations with excess body weight. These nutritional data also align with the longitudinal Italian study by Lisso et al. 2022 [13], which documented energy intakes below LARN recommendations, as well as polyunsaturated fatty acid and multiple micronutrient inadequacies across pregnancy, emphasizing persistent gaps between dietary guidelines and real-world practice.

In addition, principal component analysis identified four dietary patterns, showing distinct associations with maternal biochemical profiles and perinatal outcomes, highlighting the complex role of maternal diet in modulating maternal blood markers and placental pathways.

Interestingly, Pattern 1 (high plant, low animal: “Prudent-style”), which reflected a plant-forward dietary pattern characterized by higher adherence to legumes, nuts, fruits, vegetables, and low consumption of sauces, was positively associated with gestational age at delivery, supporting its potential role in prolonging pregnancy duration and reducing the risk of early delivery. Pattern 1 was also negatively associated with neonatal head circumference, which may suggest more favorable intrauterine growth trajectories. These observations reinforce the well-established role of plant-based, antioxidant-rich diets in supporting a healthier pregnancy trajectory and align with prior evidence indicating that “prudent-style” dietary patterns, resembling the Mediterranean diet, promote maternal metabolic health and may improve perinatal outcomes [46,80,81,82,83,84].

Conversely, Pattern 2 (ultraprocessed food, high animal), reflecting a more “Western-like” dietary profile dominated by sugars, snacks, animal fats, cereals, and dairy, was associated with higher neonatal head circumference and lower ferritin levels, suggesting potential links between pro-inflammatory dietary patterns and altered iron homeostasis and fetal growth. Moreover, despite a relatively high energy intake typical of this food profile, this pattern was not associated with improved placental efficiency, supporting the notion that energy excess in the absence of nutrient adequacy may contribute to suboptimal outcomes. Although these relationships warrant cautious interpretation, they suggest that maternal diet may subtly influence fetal growth patterns.

Among the four identified dietary patterns, Patterns 3 and 4 revealed more complex associations with maternal biomarkers and placental outcomes, reflecting the multifaceted nature of real-world dietary behaviors.

Pattern 3, characterized by high intake of fish, meat, and potatoes (high-protein, carbohydrate: “Moderately beneficial”), was positively associated with maternal total antioxidant capacity (TAC). This suggests that, despite not being classically “Prudent-style”, this pattern may include foods, such as fish, rich in omega-3 fatty acids and selenium, potentially contributing to systemic antioxidant defenses. This may represent a partially protective effect in women at metabolic risk. Moreover, no direct associations with adverse delivery outcomes were observed, suggesting a neutral or mildly protective metabolic profile. Therefore, Pattern 3 may be considered a nutritionally mixed profile with no apparent adverse associations and some indications of potential metabolic benefit.

Pattern 4, a “moderate-mixed” pattern including eggs, fruit, vegetable fats, and also non-alcoholic beverages, mostly sugar-sweetened (moderate-protein, moderate-plant, moderate-sugar), was characterized by the coexistence of food components that, when consumed in appropriate portions and frequencies, may be associated with beneficial outcomes (e.g., eggs, fruit, vegetable fats), alongside others of lower nutritional quality, such as sugar-sweetened beverages. It was associated with increased placental weight, yet with a lower neonatal-to-placental weight ratio, indicating reduced placental efficiency. This apparently paradoxical result may reflect a compensatory placental overgrowth in response to subclinical stress or nutrient imbalance, a phenomenon previously described in impaired pregnancies [9,85,86,87]. The relatively poor antioxidant profile of Pattern 4 may explain the reduced efficiency, despite its association with greater placental mass. Indeed, although the presence of fruit may contribute to micronutrient adequacy, the simultaneous intake of sugary beverages could attenuate the overall nutritional benefit, consistent with the concept of “mixed quality” dietary patterns. Interestingly, Pattern 4 was also negatively associated with maternal hemoglobin levels, possibly reflecting a suboptimal intake or bioavailability of iron within this mixed dietary profile. The presence of sugar-sweetened beverages and limited animal sources may contribute to reduced iron absorption, highlighting the importance of micronutrient adequacy during pregnancy.

It is important to acknowledge that the interpretation of mixed dietary patterns requires caution, given the coexistence of food components with potentially opposing effects. This complexity may reduce the interpretability of net associations and warrants further investigation in larger and more diverse study populations.

Nonetheless, our interpretations are further supported by the observation that in women adhering to Patterns 3 and 4, maternal energy intake was positively associated with neonatal/placental weight ratio and inversely associated with placental weight or area. This suggests that energy intake per se may support better placental efficiency, but only in the context of certain dietary patterns. In contrast, Pattern 1 (the healthiest, plant-forward/Mediterranean-inspired diet) showed no association between energy intake and placental metrics, possibly indicating that diet quality alone, rather than energy density, is sufficient to support optimal placental function. Conversely, in Pattern 2 (high in sugars, snacks, animal fats), no beneficial association with placental efficiency was found, despite higher energy intakes, further supporting the notion that energy excess without nutrient adequacy may not improve, and might even impair, placental performance.

These findings align with those of the GIFt Study [29], which also identified dietary patterns associated with maternal oxidative-inflammatory biomarkers and delivery outcomes. In that multicenter cohort, a “Western” resembling dietary pattern was associated with elevated pro-inflammatory biomarkers and higher risk of gestational complications, including lower gestational age at delivery. Our findings confirm these associations, suggesting that Pattern 2 (“Western-like pattern”) may represent a nutritionally imbalanced profile. However, our additional patterns provide a nuanced understanding of how mixed or intermediate dietary behaviors (Pattern 3—“Moderately beneficial pattern”, and Pattern 4—“Moderate-mixed pattern”) may carry partial metabolic benefits but also risks, especially when sugar or processed components are included.

Interestingly, adherence to dietary patterns varied across BMI categories, although not always in the expected direction. Adherence to Pattern 1 (“Prudent-style pattern”) differed significantly among groups, with adherence decreasing as BMI increased. However, NW women were more likely to adhere (though not significantly) also to Pattern 2 (“Western-like pattern”). OB and OW women showed greater adherence to Patterns 3 and 4 (“Moderately beneficial pattern” and “Moderate-mixed pattern”). This suggests a complex and non-linear relationship between BMI and dietary behavior, where normal-weight women may exhibit polarized eating habits, either healthier or, conversely, highly processed and sugar-rich. In contrast, overweight and obese women tended to follow intermediate or mixed-quality dietary patterns, possibly reflecting efforts to improve diet without fully achieving guideline-aligned behaviors. These findings underscore the need for tailored nutritional counseling in pregnancy, based not only on pregestational BMI, but also on actual dietary quality and pattern adherence.

Together, these results reinforce the notion that inadequate adherence to nutritional guidelines during pregnancy is widespread, even in developed countries with access to healthcare and nutritional resources.

Importantly, our study further stratifies these observations by pregestational BMI and dietary pattern, supporting the hypothesis that both maternal pregestational BMI and dietary quality during pregnancy, more than total energy intake alone, play a critical role in modulating the oxidative-inflammatory balance and optimizing maternal and fetal health [29,88]. Moreover, these results highlight that not all deviations from “Prudent-style” patterns are metabolically equivalent: while Pattern 3 may offer partial protective effects, Pattern 4 displays elements of nutritional imbalance that may influence placental development and function. The variability in dietary adherence across BMI categories further emphasizes the need for individualized nutritional strategies in pregnancy.

Our findings are consistent with growing research advocating for dietary pattern-based counseling in pregnancy, especially in women with overweight or obesity, and highlight the importance of early, targeted nutritional interventions and personalized counseling, particularly in vulnerable subgroups [13,89,90,91,92].

### 4.3. Strengths and Limitations of the Study

This study presents several strengths. It is one of the few investigations to integrate dietary pattern analysis with inflammatory and oxidative biomarkers, as well as placental and neonatal outcomes, in a well-characterized cohort of pregnant women stratified by pregestational BMI. A carefully selected cohort was enrolled, with strict exclusion criteria applied to minimize potential confounding factors, including maternal ethnicity and any maternal, fetal, or pregnancy-related pathologies. This approach was intended to isolate and better assess the specific impact of pre-pregnancy BMI and dietary patterns on molecular, biochemical, and clinical parameters. The prospective design, standardized timing of biological sample collection (third trimester and delivery), and the use of validated tools (e.g., FFQ, biochemical assays, LARN references) enhance the robustness of the findings. Moreover, the investigation of maternal biomarkers, along with dietary patterns, placental data, and neonatal outcomes, offers a holistic view of the maternal–fetal environment.

Nonetheless, some limitations must be acknowledged. The relatively small sample size—particularly in the obese subgroup—may have limited the statistical power to detect subtle differences. Dietary data were self-reported, introducing potential recall or misreporting bias, although the FFQ was adapted and validated for the Italian population. Additionally, oxidative and inflammatory biomarkers were not available for all participants due to sample availability, potentially introducing selection bias. To validate these results, future investigations should include the analysis of additional markers of oxidative stress and inflammation, in a larger population. Furthermore, although several inflammatory and oxidative stress biomarkers showed statistically significant associations with maternal BMI, the magnitude of their effect on obstetric outcomes was relatively limited. Nevertheless, these findings, while potentially lacking immediate clinical impact, offer important insights into the underlying mechanisms linking maternal adiposity to fetal development.

Notably, given the observational nature of this study, it is not possible to determine whether the associations observed reflect underlying cause–effect mechanisms. While the findings suggest potential relationships, they should be interpreted as exploratory and not as definitive proof of directional influence. As a secondary analysis, another limitation is the absence of a priori sample size calculation, which may lead to an underpowered analysis to detect certain associations. Finally, although multivariate models adjusted for major confounders, unmeasured factors (e.g., physical activity, psychosocial stress) could still influence the associations observed.

## 5. Conclusions

In conclusion, this study highlights the intertwined role of pregestational BMI and dietary quality in shaping maternal oxidative-inflammatory status and influencing placental and neonatal outcomes. By integrating clinical, biochemical, and nutritional data, we show that not only excess adiposity but also suboptimal or unbalanced dietary patterns can contribute to metabolic stress during pregnancy. While adherence to “Prudent-style” (plant-based) diets appears beneficial, Western-like or mixed patterns, even when energy intake is adequate, may still carry risks possibly affecting pregnancy progression. These findings reinforce the importance of early, personalized nutritional strategies that go beyond calorie counts to consider overall dietary composition, particularly in women with overweight or obesity, representing a strategy to improve maternal health trajectories and fetal programming. Tailored nutritional counseling should be considered a core component of antenatal care, contributing to evidence-based recommendations for optimal maternal and neonatal outcomes.

Future studies in larger, more diverse cohorts are warranted to validate these results and inform public health strategies aimed at optimizing maternal–fetal health through tailored dietary interventions.

## Figures and Tables

**Table 1:** Maternal, placental, and neonatal data.

		NW	OW	OB
Maternal General Data				
Age ^2^ [years]		34 (31–36)	34.0 (32.0–36.5)	35.0 (32.0–37.5)
Pre-pregnancy Weight ^2^ [kg]		57 (53–61)	73 (69.3–76.5) °°°	87 (81–102) °°°^,^**
Pre-pregnancy BMI ^2^ [kg/m^2^]		20.3 (19.5–22.6)	26.8 (25.8–28.2) °°°	32.3 (31.2–35.9) °°°^,^**
Supplementation:	none (%)	14.1	25.0	25.0
	only folic acid (%)	16.7	9.1	7.1
	iron (%)	14.1	11.4	25.0
	multivitamin (%)	55.1	54.5	42.9
Data at Third Trimester (T0)				
Gestational Age ^2^ [weeks]		32.0 (30.5–33.7)	31.7 (30.9–33.1)	33.0 (30.9–35.0)
Mat—GWG ^2^ [kg]		9.1 (8.0–11.0)	9.0 (7.0–12.0)	5.0 (−0.5–9.4) °°°^,^***
Mat—Hemoglobin ^1^ [g/dL]		11.6 (11.1–12.2)	11.4 (10.7–11.9)	12.1 (11.6–12.7) *
Anemia (%)		20.3	27.9	18.5
Mat—Hematocrit ^1^ [%]		34.7 (33.2–36.1)	34.0 (32.0–35.8)	36.5 (34.2–38.0) ***
Mat—Glycemia ^1^ [mg/dL]		75.0 (69.3–79.8)	79.0 (75.0–83.0) °°	80.7 (77.0–84.5) °°°
Mat—Vitamin D ^2^ [ng/mL]		23.9 (15.9–31.9)	22.0 (18.0–29.2)	23.0 (18.0–37.9)
Mat—Ferritin ^2^ [ng/mL]		11.0 (7.8–15.3)	13.0 (8.0–18.0)	13.7 (9.5–22.0)
Data at Delivery (T1)				
Gestational Age ^2^ [weeks]		39.9 (38.7–40.6)	39.7 (38.8–40.7)	39.3 (39.0–40.0)
Mat—Weight ^2^ [kg]		69.0 (63.5–75.0)	85.0 (79.1–92.0) °°°	96.0 (89.0–107.8) °°°^,^*
Mat—GWG ^2^ [kg]		12.0 (10.0–14.0)	10.3 (9.0–16.0)	7.5 (3.0–12.5) °°°^,^*
Mat—GWG according to IOM recommendations: (Chi-square test: *p*-value = 0.002)				
below (%)		37.3	15.4	28.6
within (%)		50.7	43.6	32.1
above (%)		12.0	41.0	39.3
Mat—Hemoglobin ^1^ [g/dL]		11.7 (11.1–12.3)	11.2 (10.3–12.3)	12.1 (11.2–13.1) *
Anemia (%) (Chi-square test: *p*-value = 0.038)		20.0	43.8	20.7
Mat—Hematocrit ^1^ [%]		34.9 (33.0–36.8)	33.0 (30.6–35.8)	36.0 (33.0–38.1) *
Mode of delivery:				
Spontaneous or Vacuum-Assisted delivery (%)		84.6	77.3	79.3
Cesarean Section (%)		15.4	22.7	20.7
Placental Weight ^1^ [g]		480.0 (382.5–560.0)	520.0 (430.0–609.8)	500.0 (419.5–640.0)
Placental Area ^1^ [cm^2^]		251.3 (197.9–282.7)	238.8 (201.5–274.5)	223.8 (179.5–253.7)
Neonatal/Placental Weight Ratio ^2^		6.9 (6.0–8.4)	6.6 (5.7–7.4)	6.5 (5.6–7.6)
Neonatal Weight ^2^ [g]		3340 (3135–3530)	3435.0 (3107.5–3703.8)	3380 (3190–3725)
Neonatal Head Circumference ^2^ [cm]		34.5 (33.5–35.0)	34.5 (33.5–35.0)	35.0 (33.6–35.5)
Neonatal Ponderal Index ^2^ [g/cm^3^]		2.6 (2.5–2.8)	2.6 (2.4–2.8)	2.8 (2.5–2.9)
Neonatal Sex:	Male (%)	47.4	56.8	58.6
	Female (%)	52.6	43.2	41.4
Umbilical Artery pH		7.28 (7.23–7.34)	7.24 (7.16–7.34)	7.27 (7.20–7.32)

Data were analyzed according to their distribution with ^1^ one-way ANOVA (Tukey’s HSD as post hoc test) or ^2^ Kruskal–Wallis test (pairwise comparison as post hoc test with Bonferroni correction). Data are presented as median (25th–75th percentile). Post hoc test: °° *p* < 0.01, °°° *p* < 0.001 versus NW; * *p* < 0.05, ** *p* < 0.01, *** *p* < 0.001 versus OW. BMI = Body Mass Index; Mat = Maternal; GWG = Gestational Weight Gain; IOM = Institute Of Medicine.

**Table 2:** Maternal inflammatory/oxidative biomarkers at III trimester in plasma/serum.

	NW	OW	OB
Hepcidin ^2^ [ng/mL]	71.84 (60.86–79.24) (*n* = 26)	81.40 (73.79–92.95) °° (*n* = 26)	90.38 (73.66–121.5) °°° (*n* = 18)
C Reactive Protein ^1^ [mg/L]	3.009 (1.877–4.743) (*n* = 27)	5.682 (3.733–7.090) ° (*n* = 27)	6.316 (4.339–9.528) °°° (*n* = 19)
Catalase Activity ^2^ [nmol/min/mL]	15.74 (13.27–20.21) (*n* = 28)	27.48 (18.13–42.99) °° (*n* = 25)	28.44 (23.08–49.45) °°° (*n* = 15)
SOD Activity ^1^ [U/mL]	0.600 (0.525–0.770) (*n* = 32)	0.580 (0.498–0.665) (*n* = 26)	0.620 (0.360–0.750) (*n* = 17)
TAC ^1^ [mM]	1.000 (0.963–1.028) (*n* = 28)	0.990 (0.960–1.033) (*n* = 26)	1.040 (0.985–1.065) (*n* = 17)
DNA/RNA Oxidative Damage ^2^ [pg/mL]	8108.7 (6417.3–10,156.9) (*n* = 76)	9597.8 (7601.9–13,013.4) ° (*n* = 40)	11,213.5 (9640.2–16,888.6) °°° (*n* = 23)

Data were analyzed according to their distribution with ^1^ one-way ANOVA (Tukey’s HSD as post hoc test) or ^2^ Kruskal–Wallis test (pairwise comparison as post hoc test with Bonferroni correction). Data are presented as median (25th–75th percentile). Post hoc test: ° *p* < 0.05, °° *p* < 0.01, °°° *p* < 0.001 versus NW. SOD = SuperOxide Dismutase; TAC = Total Antioxidant Capacity.

**Table 3:** Significant associations between maternal markers at 3rd trimester (T0) and clinical, neonatal/placental data.

	Maternal/General Data	β (95% CI) *p*-Value	Neonatal/Placental Data	β (95% CI) *p*-Value
Hepcidin [ng/mL]	Pregestational BMI [kg/m^2^]	β = 1.419 (0.398; 2.439) *p* = 0.006	Neon. ponderal index [g/cm^3^]	β = 0.006 (0.002; 0.009) *p* = 0.004
C Reactive Protein [mg/L]	Pregestational BMI [kg/m^2^]	β = 0.297 (0.159; 0.435) *p* = 0.000	Neon. head circumference [cm]	β = −0.135 (−0.240; −0.030) *p* = 0.012
Catalase activity [nmol/mL]	Pregestational BMI [kg/m^2^]	β = 1.536 (0.702; 2.369) *p* = 0.000	Neonatal weight [g]	β = 5.767 (0.726; 10.81) *p* = 0.025
	Gestational age T0 [weeks]	β = 2.366 (0.562; 4.171) *p* = 0.010	Placental weight [g]	β = 2.642 (0.424; 4.861) *p* = 0.020
SOD activity [U/mL]			Neon. ponderal index [g/cm^3^]	β = 0.439 (0.026; 0.851) *p* = 0.037
TAC [mM]	Multivitamin supplementation	β = 0.046 (0.010; 0.082) *p* = 0.012		
DNA/RNA oxidative damage [pg/mL]	Maternal age [years]	β = −353.5 (−652.7; −54.2)) *p* = 0.021		
	Pregestational BMI [kg/m^2^]	β = 409.9 (227.4; 592.3) *p* = 0.000		
	Folic acid supplementation	β = 4452.7 (1210.3; 7695.0) *p* = 0.007		
	Gestational age T0 [weeks]	β = 512.3 (62.5; 962.1) *p* = 0.026		

The model includes adjustment for potential confounders (maternal age, pregestational BMI, parity, supplementation; gestational age at 3rd trimester, gestational weight gain at 3rd trimester; gestational weight gain at delivery; gestational age at delivery; neonatal sex). Effect estimates indicate the amount of change in the dependent variable for every unit increase in the independent variable. Neon. = Neonatal; SOD = SuperOxide Dismutase; TAC = Total Antioxidant Capacity.

**Table 4:** Energy and macro- and micronutrient daily intakes, from FFQ.

	NW (*n* = 65)	OW (*n* = 44)	OB (*n* = 26)
Energy (kcal) ^2^	2054.7 (1644.3–2607.1)	2211.3 (1770.3–2667.4)	1860.1 (1658.2–2255.1)
Total protein (g) ^1^	86.79 (68.35–99.75)	91.54 (74.27–107.30)	84.42 (72.73–107.00)
Animal protein (g) ^1^	49.77 (38.44–65.51)	54.92 (41.29–66.70)	52.48 (42.78–75.75)
Vegetable protein (g) ^1^	35.60 (27.50–40.80)	34.50 (28.80–45.33)	31.85 (27.55–39.05)
Total carbohydrates (g) ^2^	272.8 (207.5–361.4)	293.5 (228.9–328.2)	238.8 (215.8–329.2)
Glucose (g) ^2^	12.35 (8.17–19.61)	9.60 (5.68–16.07)	10.72 (4.84–13.01)
Fructose (g) ^2^	15.70 (9.99–23.12)	11.24 (7.22–20.74)	11.80 (7.64–15.78)
Fiber (g) ^2^	29.40 (22.18–36.92)	26.69 (22.33–39.31)	23.15 (18.81–30.45)
Total lipids (g) ^2^	78.90 (61.91–99.51)	83.49 (74.09–107.13)	66.82 (56.40–91.03) *
Animal lipids (g) ^2^	38.98 (29.31–48.00)	39.34 (31.06–50.27)	32.01 (24.55–49.25)
Vegetable lipids (g) ^2^	36.38 (28.00–47.83)	43.72 (36.46–54.20) °	34.54 (27.95–44.33) *
SFA (g) ^2^	24.62 (19.93–32.35)	25.46 (20.09–33.45)	21.45 (16.15–30.68)
Arachidic acid (g) ^2^	0.17 (0.10–0.20)	0.20 (0.17–0.22) °°	0.20 (0.10–0.20)
MUFA (g) ^2^	32.17 (24.84–39.26)	36.69 (31.01–41.71)	29.80 (24.75–36.58) *
Oleic acid (g) ^2^	30.64 (23.69–37.01)	35.00 (29.66–40.09)	27.90 (22.80–34.70) *
PUFA (g) ^2^	11.42 (9.09–14.85)	12.04 (8.87–17.25)	10.25 (7.50–15.25)
EPA (g) ^2^	0.20 (0.10–0.26)	0.20 (0.10–0.30)	0.16 (0.06–0.30)
DHA (g) ^2^	0.30 (0.20–0.43)	0.30 (0.20–0.50)	0.30 (0.10–0.50)
Cholesterol (mg) ^2^	267.7 (191.3–319.2)	280.4 (223.4–358.2)	233.2 (199.7–298.9)
Iron (mg) ^2^	14.50 (11.55–18.97)	16.17 (12.15–19.89)	13.79 (10.98–19.85)
Calcium (mg) ^2^	832.1 (704.3–1105.6)	946.2 (740.4–1261.8)	855.2 (643.5–1079.8)
Sodium (mg) ^2^	1980.9 (1598.1–2434.9)	2205.7 (1717.8–2634.2)	1905.4 (1515.5–2629.0)
Potassium (mg) ^2^	3372.1 (2878.8–4268.9)	3731.9 (2782.4–4892.9)	3231.5 (2672.1–3842.3)
Phosphorus (mg) ^2^	1521.6 (1264.0–1781.7)	1648.3 (1273.1–1966.9)	1372.9 (1193.2–1754.5)
Zinc (mg) ^2^	17.14 (12.97–22.40)	20.05 (15.45–24.80)	16.37 (13.71–21.52)
Magnesium (mg) ^2^	163.3 (124.5–220.6)	167.0 (124.6–190.2)	141.8 (107.3–173.7)
Copper (mg) ^2^	1.00 (0.74–1.35)	1.06 (0.80–1.30)	0.85 (0.65–1.13)
Selenium (µg) ^2^	47.8 (37.29–61.96)	49.14 (38.41–78.48)	49.90 (33.61–69.13)
Chlorine (mg) ^2^	1245.2 (926.2–1680.3)	1241.5 (917.1–1852.2)	1135.9 (796.3–1712.1)
Iodine (µg) ^2^	92.20 (64.61–142.9)	95.30 (76.69–132.3)	97.42 (68.91–128.9)
Manganese (mg) ^2^	0.99 (0.54–1.61)	0.80 (0.42–1.78)	0.69 (0.38–1.05)
Sulfur (mg) ^1^	430.5 (342.4–544.3)	484.6 (334.0–577.5)	462.1 (315.2–598.1)
Vitamin B1 (Thiamine) (mg) ^2^	1.37 (1.15–1.86)	1.46 (1.19–1.90)	1.40 (1.15–1.95)
Vitamin B2 (Riboflavin) (mg) ^2^	1.81 (1.51–2.22)	1.95 (1.50–2.49)	1.80 (1.60–2.42)
Vitamin C (mg) ^2^	203.9 (146.0–272.0)	213.8 (122.9–286.4)	178.3 (119.3–265.4)
Niacin (mg) ^2^	20.29 (16.60–25.08)	21.85 (16.82–27.37)	20.11 (16.80–28.98)
Vitamin B6 (mg) ^2^	5.25 (3.90–6.67)	5.60 (3.87–7.33)	6.19 (5.07–7.83)
Folate (total) (µg) ^2^	338.0 (281.7–448.8)	371.1 (269.7–526.2)	335.5 (279.6–429.4)
Pantothenic acid (mg) ^2^	2.70 (1.94–3.40)	3.01 (2.21–3.63)	3.04 (2.00–3.85)
Biotin (µg) ^2^	18.80 (14.59–29.39)	22.80 (14.38–28.25)	19.50 (13.02–25.53)
Vitamin B12 (µg) ^2^	8.09 (6.13–11.34)	8.74 (6.63–13.21)	8.55 (5.00–15.08)
Vitamin A (RE) (µg) ^2^	1534.3 (1073.0–19.09.5)	1861.8 (1298.2–2145.7)	1228.9 (935.9–2092.0)
Vitamin E (α-TE) (mg) ^2^	12.75 (10.55–16.15)	15.03 (11.78–17.66)	11.48 (9.69–14.64) **
Vitamin D (µg) ^2^	2.86 (1.99–3.75)	2.62 (2.05–5.08)	2.50 (1.67–4.08)
Vitamin K (µg) ^2^	17.80 (8.81–35.71)	9.75 (4.48–21.44) °	6.55 (3.06–14.03) °°°
Water (g) ^2^	1129.7 (944.1–1441.8)	1117.9 (905.8–1549.3)	1018.4 (868.9–1251.0)

Data were analyzed according to their distribution with ^1^ one-way ANOVA (Tukey’s HSD as post hoc test) or ^2^ Kruskal–Wallis test (pairwise comparison as post hoc test with Bonferroni correction). Data are presented as median (25th–75th percentile). Post hoc test: ° *p* < 0.05, °° *p* < 0.01, °°° *p* < 0.001 versus NW; * *p* < 0.05, ** *p* < 0.01 versus OW. SFA = saturated fatty acids; MUFA = monounsaturated fatty acids; PUFA = polyunsaturated fatty acids; EPA = eicosapentaenoic acid; DHA = docosahexaenoic acid; RE = retinol equivalents; α-TE = alpha-tocopherol equivalents.

**Table 5:** Dietary patterns, from FFQ.

	Pattern 1	Pattern 2	Pattern 3	Pattern 4
Explained variance	19.1%	12.3%	9.5%	8.1%
Adhering women	54.7% of NW ^1^	50.0% of NW	32.8% of NW	48.4% of NW
	36.4% of OW ^1^	43.2% of OW	43.2% of OW	50.0% of OW
	26.9% of OB ^1^	30.8% of OB	53.8% of OB	30.8% of OB
Adherence value ^2^:				
NW	0.495 (0.243–1.054)	0.700 (0.364–1.346)	0.442 (0.232–0.794)	0.340 (0.118–0.990))
OW	0.726 (0.431–1.477)	0.657 (0.246–1.214)	0.746 (0.132–1.920)	0.805 (0.366–1.603) °
OB	0.701 (0.326–1.224)	0.352 (0.067–1.318)	0.635 (0.280–1.540)	0.530 (0.382–0.784)
Food categories:				
Dairy	−0.075	**0.481**	0.186	0.192
Cereals	0.384	**0.529**	0.131	0.139
Vegetables	**0.596**	−0.045	0.277	0.371
Legumes	**0.713**	0.213	0.123	−0.124
Potatoes	0.071	0.217	**0.608**	−0.038
Meat	−0.304	0.253	**0.694**	0.093
Fish	0.233	−0.220	**0.772**	0.089
Eggs	−0.126	−0.056	0.031	**0.715**
Fruits	**0.395**	0.214	0.064	**0.545**
Nuts	**0.611**	0.000	−0.076	0.161
Vegetable fats	−0.005	0.186	0.287	**0.503**
Animal fats	0.017	**0.674**	−0.060	−0.292
Sauces	**−0.452**	0.319	0.226	0.120
Sugars and snacks	0.044	**0.735**	0.054	0.273
Non-alcoholic beverages	0.215	0.089	−0.115	**0.494**

Relation between food categories and dietary patterns, expressed by factor loadings. The factor loadings indicate how much each food category correlates with the extracted dietary patterns. The factor loadings with the highest absolute value, highlighted in bold type, were used for pattern labelling. Adherence values are presented as median (25th–75th percentile). ^1^ Chi Square Test, *p* < 0.05; ^2^ Kruskal–Wallis test (pairwise comparison as post hoc test with Bonferroni correction). Post hoc test: ° *p* < 0.05 versus NW. Please note: Each woman did not exclusively adhere to a single pattern but might adhere to more than one.

**Table 6:** Significant associations between adherence to dietary patterns, maternal blood markers at 3rd trimester, and neonatal and placental data.

	3rd Trimester Maternal Blood Markers	β (95% CI) *p*-Value	Neonatal and Placental Data	β (95% CI) *p*-Value
Pattern 1			Gestational age at T1 [weeks]	β = 0.243 (0.019; 0.466) *p* = 0.033
			Neon. head circumference [cm]	β = −0.414 (−0.826; −0.001) *p* = 0.050
Pattern 2	Ferritin [ng/mL]	β = −2.093 (−4050; −0.135) *p* = 0.036	Neon. head circumference [cm]	β = 0.403 (0.026; 0.779) *p* = 0.036
Pattern 3	TAC [mM]	β = 0.015 (0.000; 0.030) *p* = 0.046		
Pattern 4	Hemoglobin [g/dL]	β = −0.223 (−0.402; −0.043) *p* = 0.015	Placental weight [g]	β = 31.479 (7.723; 55.235) *p* = 0.009
			Neonatal/Placental weight ratio	β = −0.384 (−0.717; −0.052) *p* = 0.023
In Pattern 3 Energy [kcal]			Placental area [cm^2^]	β = −0.031 (−0.058; −0.005) *p* = 0.020
			Neonatal/Placental weight ratio	β = 0.001 (0.000; 0.001) *p* = 0.044
In Pattern 4 Energy [kcal]			Placental weight [g]	β = −0.041 (−0.081; −0.000) *p* = 0.049
			Placental area [cm^2^]	β = −0.027 (−0.051; −0.003) *p* = 0.026
			Neonatal/Placental weight ratio	β = 0.001 (0.000; 0.001) *p* = 0.040

The model includes adjustment for potential confounders (maternal age, pregestational BMI, parity, supplementation. For 3rd trimester molecular data: gestational age at 3rd trimester, gestational weight gain at 3rd trimester. For neonatal and placental data: energy; gestational age at delivery, gestational weight gain at delivery, neonatal sex). Effect estimates indicate the amount of change in the dependent variable for every unit increase in the independent variable. Neon. = Neonatal; TAC = Total Antioxidant Capacity.

## Data Availability

All data that support the findings of this study are available from the authors upon reasonable request due to privacy reasons.

## Supplementary Materials

The following supporting information can be downloaded at: https://www.mdpi.com/article/10.3390/nu17162590/s1, File S1: Example of calculation of the daily amount of energy, macro- and micronutrients.
