# Supplementary material for: Maternal BMI and Diet Quality Modulate Pregnancy Oxidative and Inflammatory Homeostasis
Source: Nutrients. 2025 Aug 9;17(16):2590. doi: 10.3390/nu17162590 (PMC12389677; doi:10.3390/nu17162590)
Supplement: Supplementary file 1 [file nutrients-17-02590-s001.zip › nutrients-3780495-supplementary.pdf]

SUPPLEMENTARY FILE S1

Example of calculation of the daily amount of energy, macro- and micronutrients

Example: consumption of a higher than standard portion of corn flakes 3-4 times per week.
The dietitian multiplied standard size portion (expressed as hg, 30g/100=0.3) by eventual portion variation (in this case, 1.25) by the corresponding daily frequency (in this case, 3.5 times / 7 days = 0.5). This product was then multiplied by the bromatological composition (energy, macro- and micronutrient items; expressed for 100g) of corn flakes: for instance, 100g corn flakes provide 6.6g of proteins, thus daily protein intake from corn flakes in this case was 0.3*1.25*0.5*6.6=1.2375g.
The same calculation was done for energy and for each macro- and micronutrient item provided by corn flakes; all these calculations were done for each food item of the FFQ.
At last, the dietitian summed the results from each food item, obtaining the daily intake of energy, macro and micronutrients.
